# Network Pharmacology Combined With Gut Microbiome and Serum Metabolomics Reveals the Therapeutic Mechanisms of Hydroxysafflor Yellow A in Diabetic Kidney Disease

**DOI:** 10.1155/jdr/2131566

**Published:** 2026-04-01

**Authors:** Pingping Wang, Xinyu Liu, Wen Sun, Xueyun Dong, Jiajun Tan, Min Chen, Jiayuan He, Asmaa Ali, Liang Wu, Keke Shao

**Affiliations:** ^1^ Department of Laboratory Medicine, Taizhou Second People’s Hospital, Taizhou, 225309, China; ^2^ Department of Laboratory Medicine, The Yancheng Clinical College of Xuzhou Medical University, The First People’s Hospital of Yancheng, Yancheng, 224000, China; ^3^ Department of Critical Care Medicine, Jurong Hospital Affiliated to Jiangsu University, Zhenjiang, 212499, China; ^4^ Department of Laboratory Medicine, School of Medicine, Jiangsu University, Zhenjiang, 212013, China, ujs.edu.cn; ^5^ Health Testing Center, Zhenjiang Center for Disease Control and Prevention, Zhenjiang, 212002, China; ^6^ Department of Pulmonary Medicine, Abbassia Chest Hospital, EMOH, Cairo, 11517, Egypt; ^7^ Molecular Medical Research Center, Yancheng Clinical Medical College of Jiangsu University, Yancheng, 224000, China

**Keywords:** AGE–RAGE axis, diabetic kidney disease, gut microbiota, hydroxysafflor yellow A, network pharmacology, oxidative stress, serum metabolomics

## Abstract

Diabetic kidney disease (DKD) is a severe complication of diabetes, primarily driven by chronic inflammation, oxidative stress, and gut microbiota dysbiosis. Hydroxysafflor yellow A (HSY), a bioactive compound derived from *Carthamus tinctorius* L., demonstrates promising renoprotective effects. However, its mechanisms, especially through modulation of the gut–kidney axis, remain poorly understood. This study employed a combination of network pharmacology, a high‐fat diet/streptozotocin‐induced type 2 diabetic mouse model, 16S rRNA sequencing, and serum metabolomics to explore the therapeutic mechanisms of HSY. Renal function, oxidative stress, inflammation, and gut microbiota composition were evaluated. HSY significantly alleviated renal injury by reducing blood glucose, creatinine, and urea nitrogen levels (*p*  < 0.05), while enhancing renal antioxidant enzyme activity (GSH, SOD, CAT). Inflammatory markers (TNF‐α, IL‐1β) and AGE–RAGE signaling were suppressed. Analysis of the gut microbiota revealed that HSY enriched SCFA‐producing genera (e.g., Lactobacillus, Alloprevotella) and decreased the abundance of Schaedlerella. Serum metabolomics further indicated that HSY modulated riboflavin metabolism, linoleic acid metabolism, and steroid hormone biosynthesis, thereby linking microbial metabolites to renal protection. Spearman correlation analysis revealed strong associations between specific gut microbiota (e.g., Prevotella) and serum metabolites (e.g., eicosapentaenoic acid). HSY mitigates DKD by targeting AGE–RAGE‐mediated inflammation, oxidative stress, and gut microbiota dysbiosis while correcting metabolic disturbances. This study offers a novel multi‐omics approach to understanding HSY’s renoprotective effects, highlighting its potential as a therapeutic agent for DKD.

## 1. Introduction

Diabetic kidney disease (DKD), a common microvascular complication of diabetes mellitus, is a leading cause of end‐stage renal disease globally [1, 2]. Chronic hyperglycemia triggers pathological processes, including oxidative stress, inflammation, and extracellular matrix accumulation, culminating in glomerulosclerosis and renal fibrosis [1, 2]. Despite advancements in glycemic control and renoprotective therapies (e.g., angiotensin‐converting enzyme inhibitors and sodium‐glucose cotransporter‐2 inhibitors), DKD progression remains poorly controlled, highlighting the urgent need for novel therapeutic strategies targeting its multifactorial pathogenesis [3–5].

Hydroxysafflor yellow A (HSY), the principal bioactive quinonechalcone glycoside (C27H32O16) isolated from *Carthamus tinctorius* L. (safflower), has emerged as a promising therapeutic agent due to its multifaceted pharmacological properties. Extensive phytochemical studies have characterized HSYA as a water‐soluble compound with a molecular weight of 612.53 g/mol, featuring a unique conjugated system that contributes to its potent antioxidant capacity [6, 7].

Growing evidence from preclinical investigations has demonstrated that HSY exerts remarkable anti‐inflammatory, antioxidant, and anti‐apoptotic effects through modulation of various signaling pathways, including NF‐κB, Nrf2/HO‐1, and PI3K/Akt [8, 9]. These mechanisms underlie its therapeutic potential in diverse pathological conditions, particularly in cardiovascular and metabolic disorders. Notably, HSYA has shown significant efficacy in ameliorating ischemia‐reperfusion injury, attenuating atherosclerotic progression, and preventing diabetic complications such as retinopathy [6, 10, 11].

Extensive toxicological studies confirm that HSYA exhibits excellent biocompatibility, with safe doses up to 200 mg/kg/day in rodents and in vitro viability >90% at concentrations below 100 μM (~61.25 μg/mL) [12]. Importantly, HSYA is predominantly renally excreted (>70% unchanged in urine within 24 h) following oral or intravenous administration, suggesting preferential kidney exposure that may enhance its therapeutic potential for diabetic renal injury [13].

Notably, HSY modulates nuclear factor‐kappa B (NF‐κB) and NOD‐like receptor family pyrin domain‐containing 3 (NLRP3) inflammasome pathways, suggesting potential renoprotective mechanisms [14, 15]. Mechanistically, HSY‐mediated NF‐κB suppression reduces pro‐inflammatory cytokine production (TNF‐α, IL‐6, IL‐1β), thereby mitigating glomerular endothelial inflammation and podocyte injury—hallmark pathologies in DKD progression. Concurrently, NLRP3 inflammasome inhibition blocks caspase‐1 activation and subsequent pyroptosis, preserving renal tubular epithelial cell integrity. Crucially, these anti‐inflammatory effects synergistically (1) maintain glomerular filtration barrier function by preventing podocyte foot process effacement, (2) attenuate tubulointerstitial fibrosis via TGF‐β/Smad3 pathway downregulation, and (3) reduce albuminuria by stabilizing nephrin expression in slit diaphragms [16].

Emerging evidence underscores the role of gut microbiota dysbiosis in DKD progression [17, 18]. Dysregulated microbial metabolites (e.g., short‐chain fatty acids, trimethylamine‐N‐oxide) exacerbate systemic inflammation and oxidative stress, contributing to renal dysfunction [19, 20]. Additionally, serum metabolomic alterations reflect the dynamic relationship between host metabolism and microbial activity, providing valuable insights into disease mechanisms [21, 22]. The integration of gut microbiota profiling with untargeted metabolomics thus offers a robust framework for investigating the systemic effects of therapeutic interventions.

This study aims to assess the efficacy of HSY in mitigating renal injury in a type 2 diabetic murine model and explore its underlying mechanisms through two key perspectives: modulation of gut microbiota and serum metabolic alterations. It is hypothesized that HSY alleviates DKD by restoring microbial homeostasis and correcting metabolic disturbances associated with oxidative stress and inflammation. These findings may position HSY as a promising multi‐target therapeutic for DKD while advancing understanding of the gut–kidney axis in diabetic complications.

## 2. Materials and Methods

### 2.1. Drug Target Prediction and Network Pharmacology Analysis

The molecular structure of HSY was retrieved from PubChem and subjected to multi‐database target prediction using SEA Search, SwissTargetPrediction, TargetSUPER, and CBligand. Potential disease‐associated targets of T2DM were screened from GeneCards, OMIM, and TTD. A Venn diagram was generated to map the common targets between HSY and T2DM.

For network analysis, the overlapping targets were uploaded to STRING to construct a protein–protein interaction (PPI) network. The resulting data in TSV format were imported into Cytoscape 3.9.0, where core therapeutic targets were identified using CytoNCA based on topology parameters (degree centrality). Functional enrichment analysis was performed via the DAVID database to elucidate biological processes, molecular functions, and KEGG pathways (*p* < 0.05), providing insights into HSY’s mechanisms against T2DM. Detailed procedures are provided in Supporting Information: S1.

### 2.2. Establishment of T2DM Mouse Model and Experimental Protocol

Male ICR mice (22–26 g; Wukong Biotechnology, Nanjing) were utilized in this study. After 1 week of acclimatization, the animals were randomly divided into four groups (*n* = 6/group): (1) Normal control (NC), (2) T2DM model, (3) HSY high‐dose (HSYH, 10 mg/kg), and (4) HSY low‐dose (HSYL, 5 mg/kg). T2DM induction was performed as previously described with modifications [23]. Briefly, 6‐week‐old mice were fed a high‐fat diet (60% kcal from fat) for 2 weeks, followed by intraperitoneal injection of streptozotocin (50 mg/kg/day; Merck) for three consecutive days. Successful model establishment was confirmed by fasting blood glucose levels ≥11.1 mmol/L at week 4. Animals continued the high‐fat regimen until week 12.

HSY treatment (Shanghai Shifeng Biological) began simultaneously with model induction and continued throughout the study period. At the experimental endpoint, mice were euthanized *via* intraperitoneal urethane injection (700 mg/kg; Sigma–Aldrich). Serum samples, colonic contents, and renal tissues were collected for subsequent analyses. Detailed procedures are provided in Supporting Information: S2.

### 2.3. Analysis of Serum Parameters and Renal Oxidative Stress Markers

Serum glucose, renal function markers (BUN and CRE), and lipid parameters (TC, TG, HDL‐C, and LDL‐C) were quantified using commercial assay kits from Jiancheng Bioengineering (Nanjing), while renal oxidative status was assessed by measuring antioxidant enzymes (T‐SOD and CAT), reduced glutathione (GSH), and advanced glycation end products (AGEs; Meimian Bioengineering) according to the manufacturers’ protocols. Detailed procedures are provided in Supporting Information: S3.

### 2.4. Quantitative Real‐Time PCR (qRT‐PCR) and Western Blot Analysis

The mRNA expression levels of TNF‐α, IL‐1β, and RAGE in renal tissue were analyzed by quantitative qRT‐PCR using commercially available kits (Vazyme, Nanjing, China). Total RNA was isolated using RNA‐easy Isolation Reagent, and complementary DNA (cDNA) was synthesized with HiScript III RT SuperMix, followed by PCR amplification using AceQ Universal SYBR qPCR Master Mix with gene‐specific primers (relative expression levels were calculated using the 2^−ΔΔCT^ method). Additionally, protein expression levels of NLRP3, Caspase‐1, and RAGE in renal tissues were assessed by Western blot, starting with tissue homogenization in RIPA lysis buffer (Beyotime, China) supplemented with protease inhibitor cocktail (MedChemExpress, USA), centrifugation at 12,000×g for 15 min at 4°C, and protein quantification using the BCA Protein Assay Kit (Beyotime). Equal protein amounts were resolved by 10% SDS‐PAGE, transferred onto PVDF membranes (Millipore, USA), blocked with 5% non‐fat milk, and probed overnight at 4°C with primary antibodies against NLRP3 (1:1000, Cell Signaling Technology), Cleaved Caspase‐1 (1:1000, Abcam), RAGE (1:1000, Proteintech), and β‐actin (1:5000, Proteintech) as the loading control. After incubation with HRP‐conjugated secondary antibodies (1:5000, Proteintech), protein bands were detected using ECL Plus Substrate (Vazyme, China), visualized via chemiluminescence imaging (Tanon, China), and quantified using ImageJ software (NIH, USA). Detailed methodologies are provided in Supporting Information: S4.

### 2.5. HE Staining, Masson Staining, and RAGE Immunohistochemical Staining

Mouse kidney tissue was fixed in 4% paraformaldehyde for 24 h, embedded in paraffin, and sectioned for HE and Masson staining to assess pathological changes and fibrosis in the kidney. Immunohistochemistry was used to evaluate RAGE expression in the kidney tissue. The immunohistochemical protocol included standard deparaffinization and washing of paraffin sections, endogenous peroxidase inhibition with 3% H_2_O_2_ in methanol, followed by blocking with 1:10 goat serum at 37°C for 20 min. Rabbit anti‐RAGE antibody was incubated overnight at 4°C, followed by the addition of a secondary antibody (1:200) and incubation at 37°C for 20 min. DAB chromogenic solution was then applied, and RAGE expression was observed under a microscope.

### 2.6. Gut Microbiota Profiling and Serum Metabolomics

Gut microbiota composition was characterized through 16S rRNA gene sequencing of colonic contents (Ekemo Tech Group Co., Ltd., Shenzhen, China). Bacterial community analysis included diversity assessments (Shannon index for α‐diversity; PCoA for β‐diversity) and heatmap visualization to evaluate structural changes induced by HSYA treatment.

For serum metabolomics, untargeted profiling was conducted using liquid chromatography‐mass spectrometry (LC‐MS). Multivariate statistical approaches (PCA and orthogonal partial least squares discriminant analysis [OPLS‐DA]) were applied to distinguish metabolic patterns between groups. Differential metabolites were initially screened using a variable importance in projection (VIP) score >1.0 (from OPLS‐DA) and unadjusted *p*  < 0.05 (Student’s *t*‐test). To control for false discoveries, *p*‐values were further adjusted using the Benjamini–Hochberg false discovery rate (FDR) correction, with FDR‐adjusted *p*  < 0.05 considered statistically significant. Kyoto Encyclopedia of Genes and Genomes (KEGG) pathway enrichment analysis was performed using MetaboAnalyst 6.0 to elucidate altered metabolic networks. Detailed procedures, including parameter settings for FDR correction, are provided in Supporting Information: S5.

### 2.7. Statistical Analysis

Statistical analyses were conducted using SPSS 20.0 (IBM Corp., Armonk, NY, USA), with continuous variables expressed as mean ± standard deviation (SD). Intergroup comparisons were performed using one‐way analysis of variance (ANOVA), followed by Tukey’s honestly significant difference (HSD) post hoc test for multiple comparisons. A two‐tailed significance threshold of *p*  < 0.05 was applied for all statistical inferences. Data visualization was carried out using GraphPad Prism 9.0 (GraphPad Software, San Diego, CA, USA), Bioincloud (www.bioincloud.tech), and RStudio (R Foundation for Statistical Computing, Vienna, Austria).

## 3. Results

### 3.1. Network Pharmacology Analysis Results of HSY in the Treatment of T2DM

The TTD database identified 98 disease‐related targets for T2DM, the OMIM database retrieved 200 disease targets, and the Genecards database yielded 1275 disease targets. After merging these three databases and removing duplicates, 1961 disease targets were obtained. The PharmMapper database identified 281 drug action targets for HSYA, the SwissTarget Prediction database found 94 targets, and the CTD database identified 32 targets. After merging and eliminating duplicates, 403 predicted targets were obtained. The intersection of predicted disease targets and drug action targets revealed 84 common targets (Figure 1A).

Figure 1Pharmacological network analysis of HSY treatment targets for T2DM. A total of 84 potential therapeutic targets were identified through analysis (A). Further screening through PPI network analysis revealed that TNF, STAT3, and IL‐6 are the main targets of HSY for treating T2DM (B, C). GO enrichment analysis (D) and KEGG analysis (E) were used to study the mechanism of HSY in treating T2DM.

**Figure:**
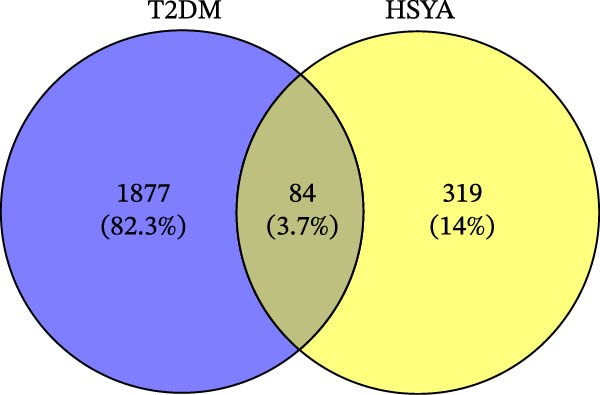
(A)

**Figure:**
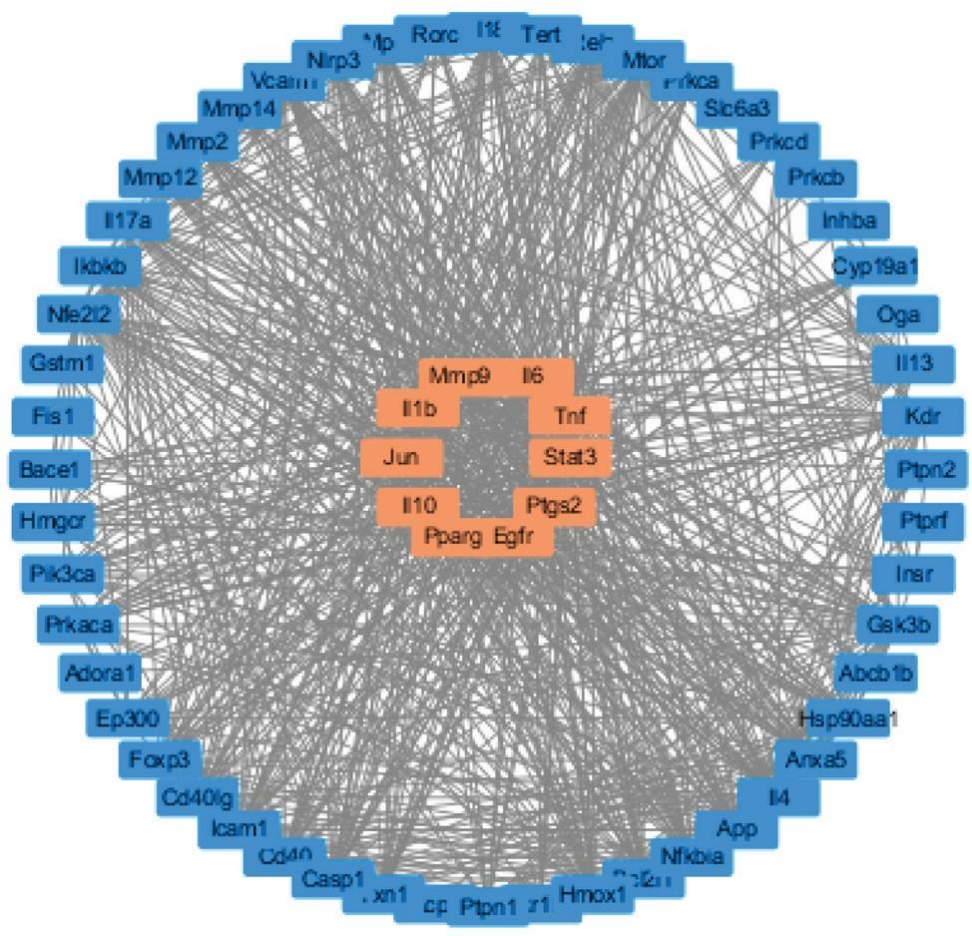
(B)

**Figure:**
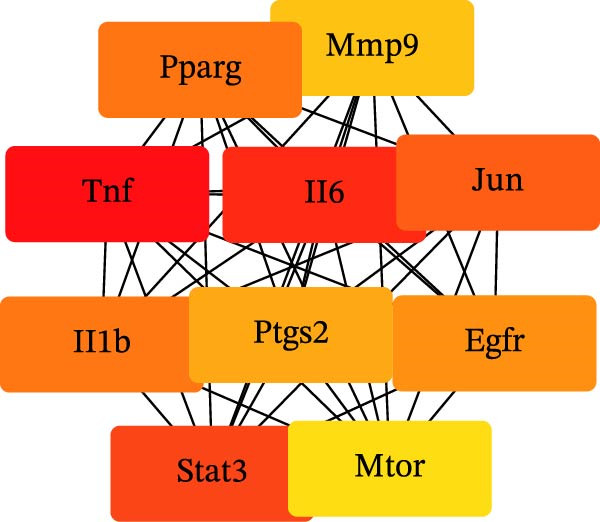
(C)

**Figure:**
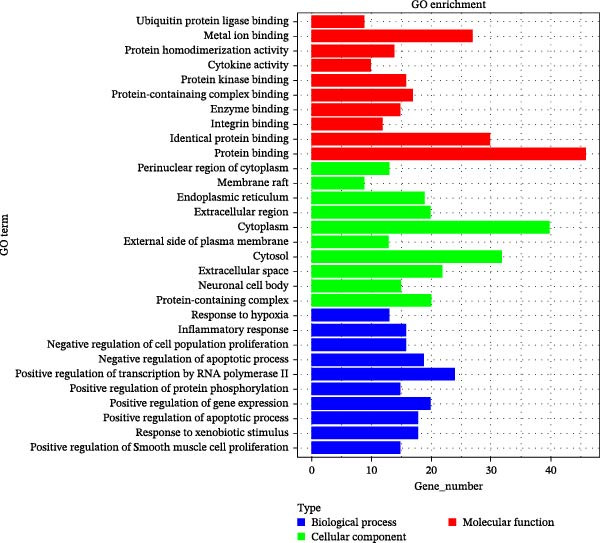
(D)

**Figure:**
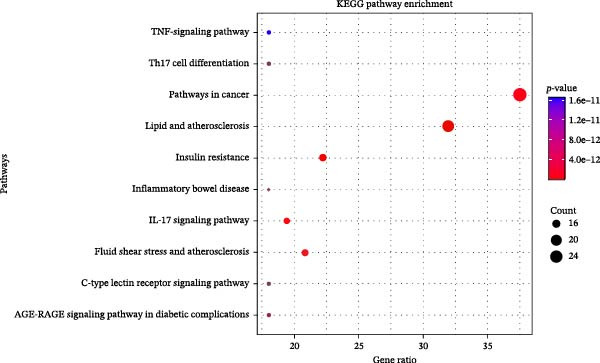
(E)

The 185 intersecting targets were imported into the String database to predict PPI relationships, constructing a PPI network (Figure 1B). Further analysis using Cytoscape software allowed for the construction of a network diagram and identification of the top 10 targets. Among these, TNF, STAT3, and IL‐6 emerged as key targets of HSYA in T2DM treatment (Figure 1C).

The potential targets of HSY for T2DM treatment underwent Gene Ontology (GO) enrichment analysis via the DAVID database, which revealed 516 biological processes, 76 GO cellular components, and 92 GO molecular functions. The biological processes primarily involved the positive regulation of smooth muscle cell proliferation, response to external stimuli, and positive regulation of apoptosis. Cellular components mainly included protein‐containing complexes, neuronal cell bodies, and extracellular spaces. Molecular functions were primarily associated with protein binding, identical protein binding, and integrin binding (Figure 1D). KEGG analysis identified 156 signaling pathways, which included lipid metabolism and atherosclerosis in diabetic complications, insulin resistance, and the AGE–RAGE signaling pathway in diabetic complications, among others (Figure 1E).

### 3.2. HSYA Significantly Improves the Serum Biochemical Indicators in T2DM Mice

In terms of animal model data, the body weight of mice in the NC group continued to increase throughout the experiment. After 3 weeks of high‐fat diet feeding and STZ induction, mice in the T2DM, HSYL, and HSYH groups experienced a significant reduction in body weight. This trend was partially reversed in the HSYL and HSYH groups following HSY intervention starting in the fourth week. From the sixth week onwards, the body weight of mice in the T2DM group remained significantly lower than in the HSYL and HSYH groups (*p*  < 0.05) (Figure 2A,B).

Figure 2Body weight and serum biochemical indicators of mice (*n* = 6). From the 6th week, the NC group mice showed significantly higher body weight compared to the T2DM, HSYH, and HSYL groups (A). By the 7th and 8th weeks, the HSYH group mice had significantly higher body weight than the T2DM group (B). At the end of the experiment, the HSYH and HSYL group mice had significantly lower levels of FBG (C), BUN (D), CRE (E), and TG (F) compared to the T2DM group. However, there was no significant improvement in the TG (G), LDL‐C (H), and HDL‐C (I) levels of the T2DM mice.  ^∗^ *p*  < 0.05.

**Figure:**
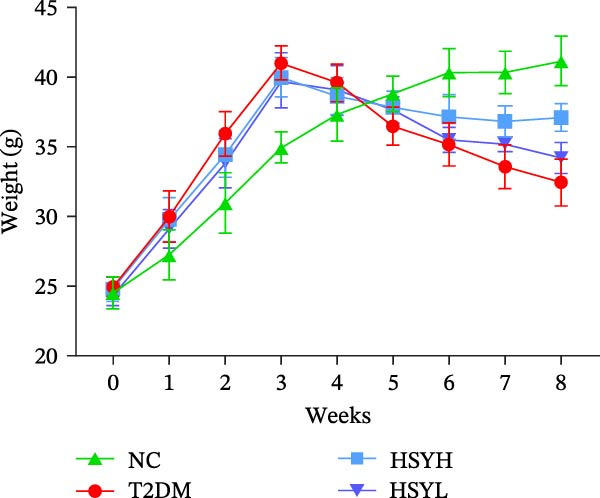
(A)

**Figure:**
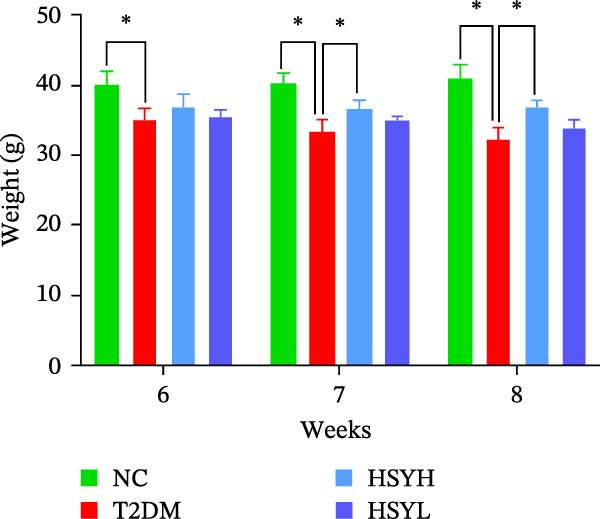
(B)

**Figure:**
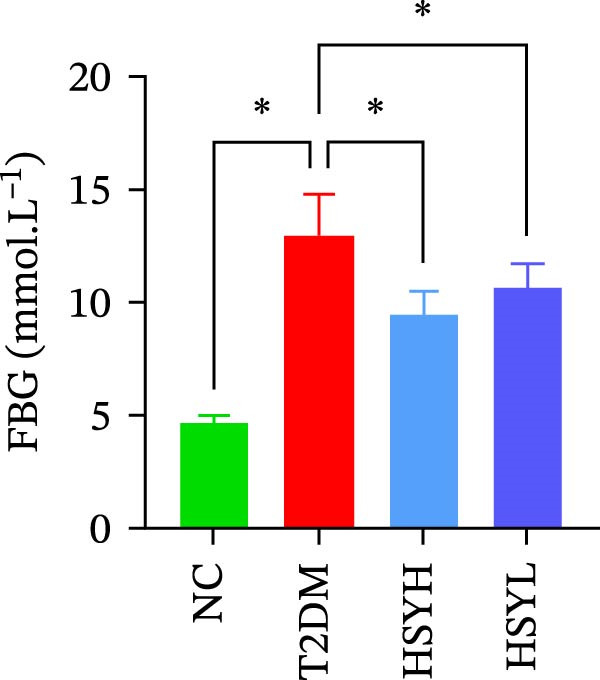
(C)

**Figure:**
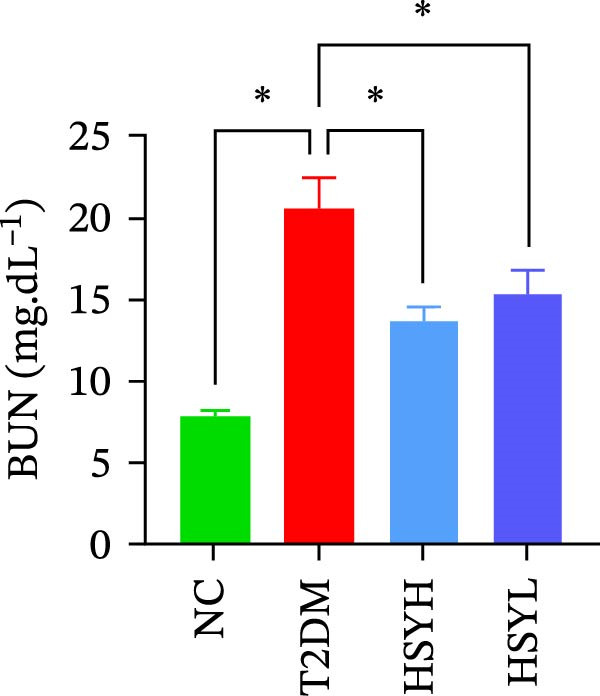
(D)

**Figure:**
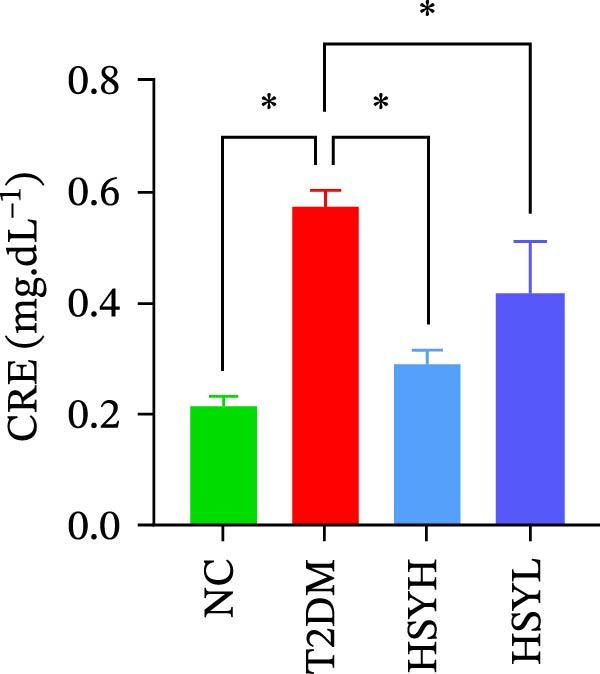
(E)

**Figure:**
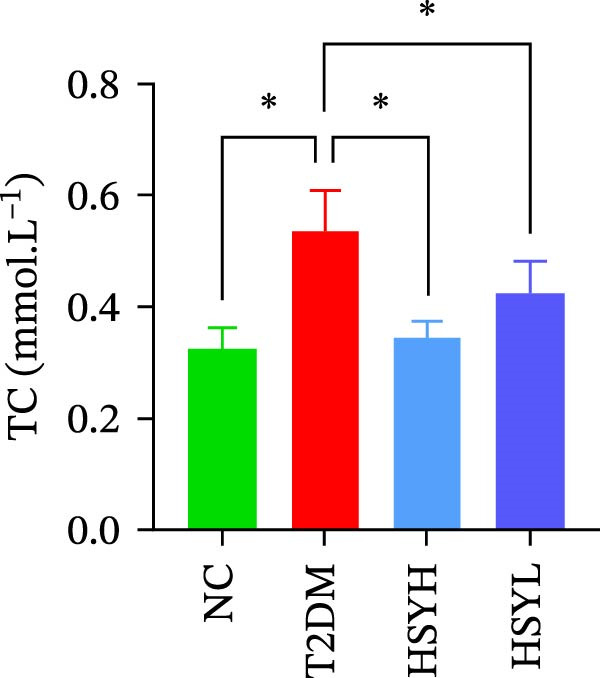
(F)

**Figure:**
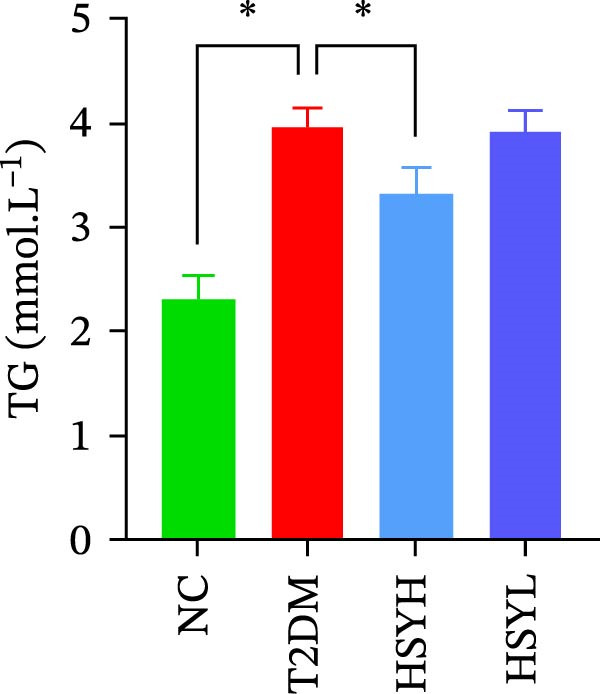
(G)

**Figure:**
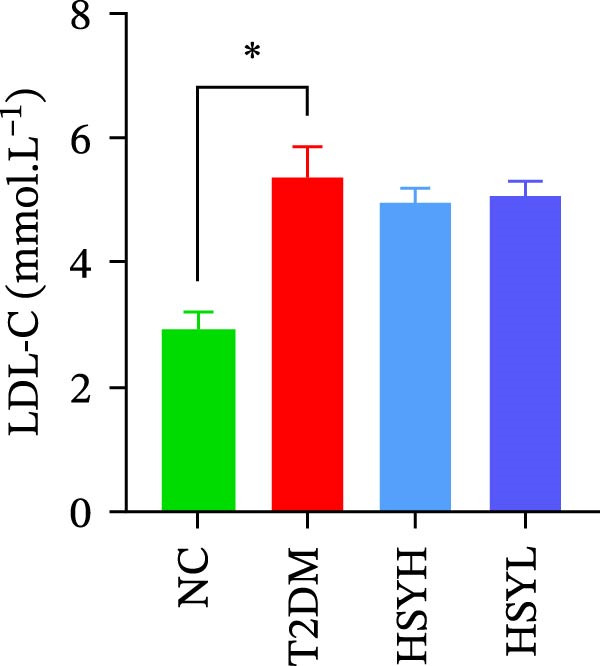
(H)

**Figure:**
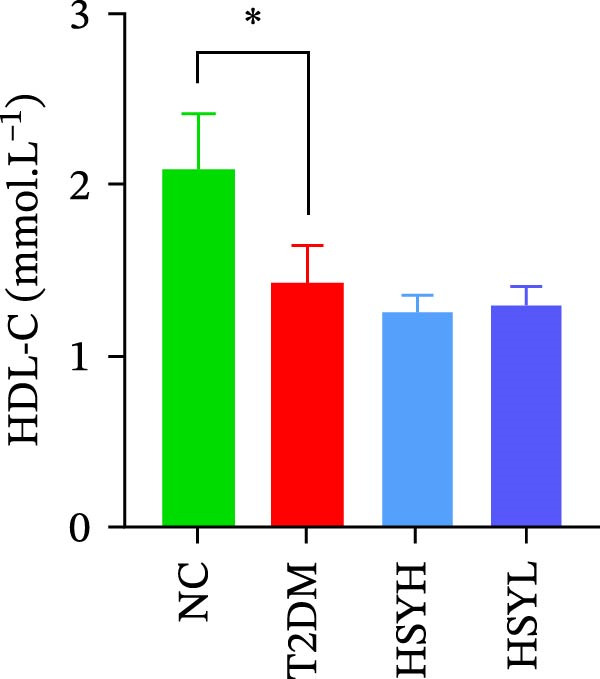
(I)

Serum levels of FBG, BUN, CRE, TC, TG, and LDL‐C were significantly higher in the T2DM group compared to the NC group (*p*  < 0.05). In contrast, the HSYL and HSYH groups showed significantly reduced levels of FBG, BUN, CRE, and TC compared to the T2DM group (*p*  < 0.05), with the high‐dose HSY group exhibiting a more pronounced improvement in these biochemical markers (Figure 2C–G). However, HSYA had no significant effect on serum LDL‐C or HDL‐C levels (*p*  > 0.05) (Figure 2H,I).

### 3.3. HSY Improved Inflammation and Oxidative Stress Responses in the Kidneys of T2DM Mice and Inhibited RAGE Expression

The levels and activities of GSH, SOD, and CAT in the kidneys of mice were assessed using chemical methods, the concentration of AGEs in serum was quantified by ELISA, the mRNA expression of TNF‐α, IL‐1β, and RAGE in kidney tissues was measured by qPCR, and the protein expression of NLRP3, Caspase‐1, and RAGE was determined by Western blot assay (Figure 3). Western blot analysis further confirmed that both high‐ and low‐dose HSY significantly suppressed the protein expression of inflammatory factors NLRP3, Caspase‐1, and RAGE in the kidneys of T2DM mice. Compared to the NC group, the T2DM group showed significantly reduced levels/activities of oxidative stress factors GSH, SOD, and CAT in the kidneys. Additionally, mRNA expression levels of TNF‐α, IL‐1β, and RAGE were significantly increased, and serum AGE concentrations were notably elevated (*p*  < 0.05). High concentrations of HSY, in comparison to the T2DM group, significantly restored the levels/activities of oxidative stress factors GSH, SOD, and CAT in the kidneys (*p*  < 0.05). Moreover, HSY treatment reduced the expression levels of pro‐inflammatory factors TNF‐α, IL‐1β, and RAGE in the kidneys and lowered serum AGE levels (*p*  < 0.05).

Figure 3Inflammation and oxidative stress indicators in mouse kidneys, as well as serum AGEs levels (*n* = 6). Biochemical assay kits were used to measure the concentration/activity of GSH (A), SOD (B), and CAT (C) in mouse kidney tissue, and an ELISA kit was used to measure serum AGEs concentration (G). qPCR was used to detect the expression levels of TNF‐α (D), IL‐1β (E), and RAGE (F) in kidney tissue. Western blot assay was used to detect the expression of NLRP3, Caspase‐1 and RAGE (H)  ^∗^ *p*  < 0.05.

**Figure:**
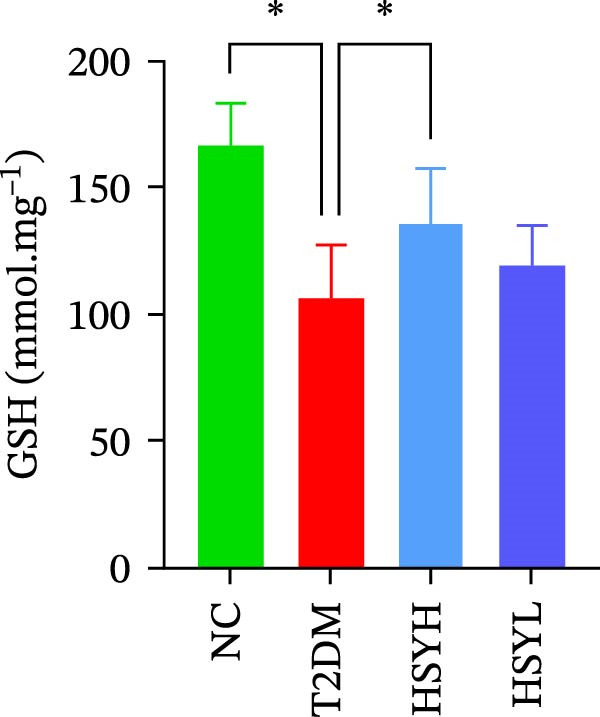
(A)

**Figure:**
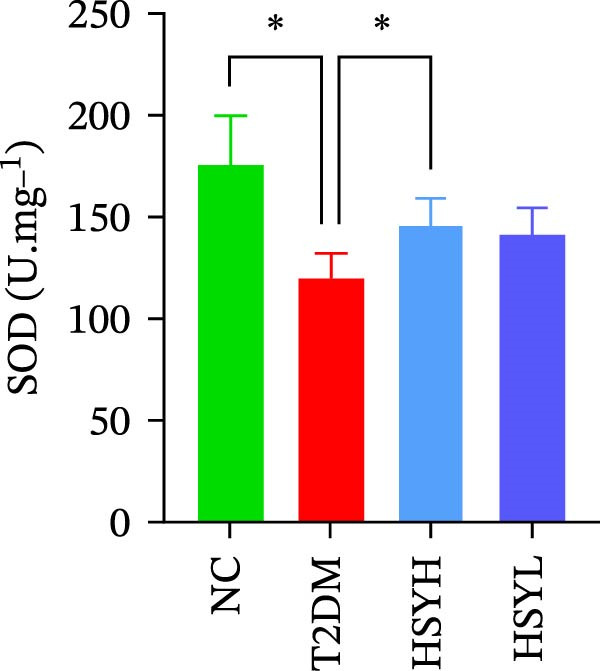
(B)

**Figure:**
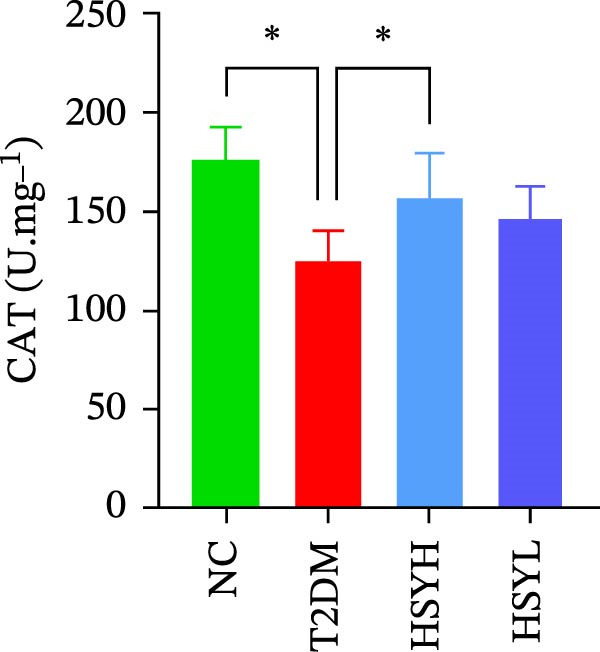
(C)

**Figure:**
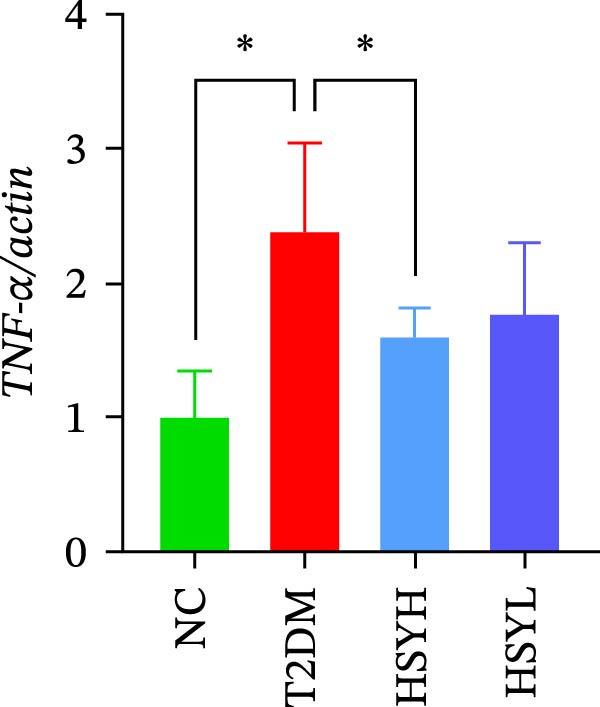
(D)

**Figure:**
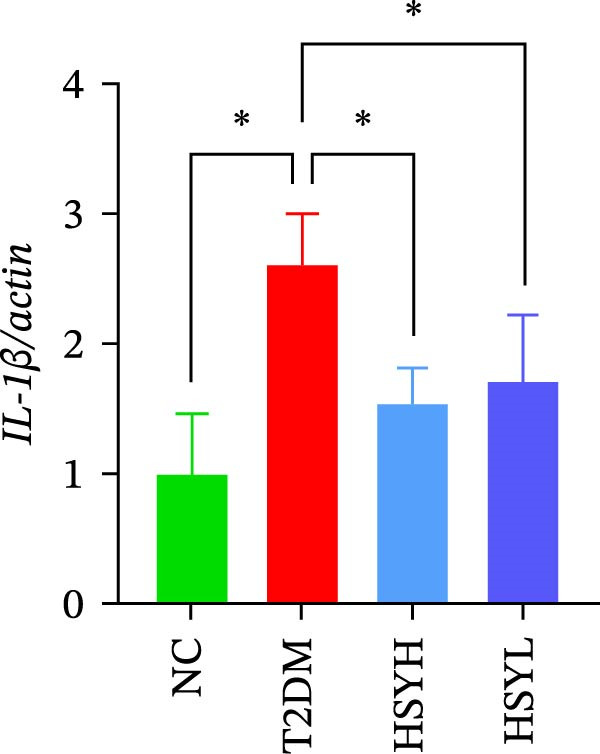
(E)

**Figure:**
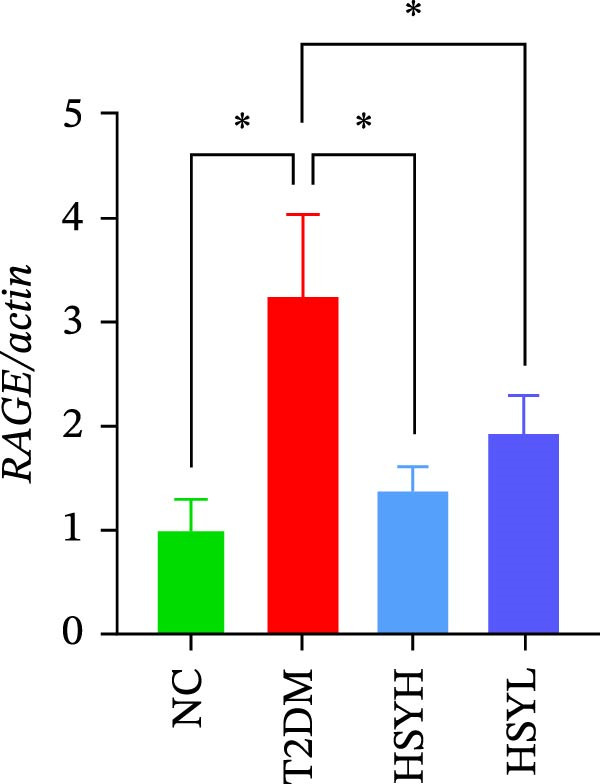
(F)

**Figure:**
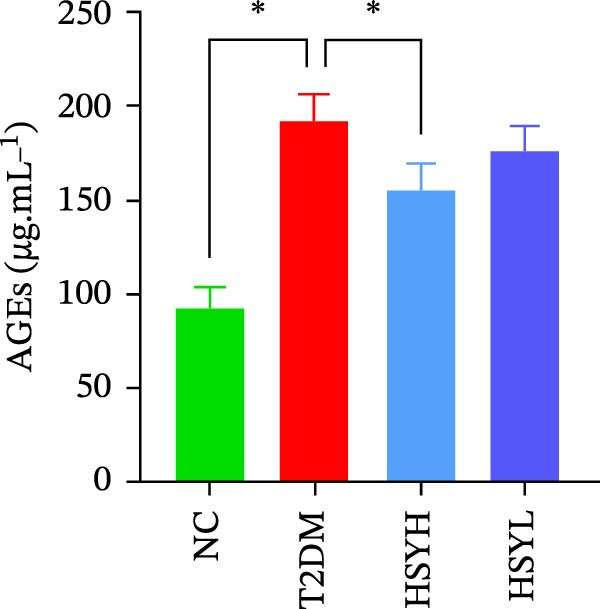
(G)

**Figure:**
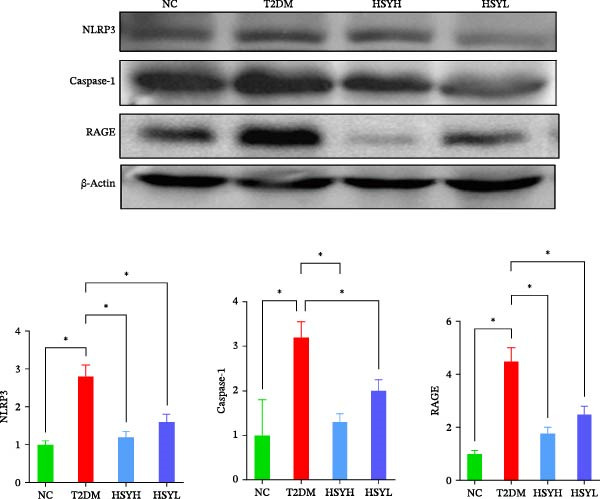
(H)

### 3.4. Histopathological Analysis of Mouse Kidneys

**Figure 4:**
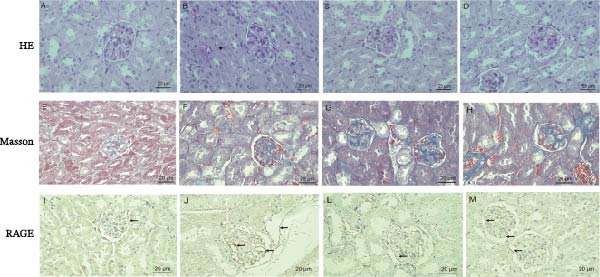
HE staining of mouse kidneys (A–D), Masson staining (E–H), and RAGE immunohistochemical staining (I–M) (*n* = 3). The NC group includes A, E, and I; the T2DM group includes B, F, and J; the HSYH group includes C, G, and L; the HSYH group includes D, H, and M.

Histological examination using HE staining revealed that in the NC group, the glomeruli were of uniform size, without mesangial proliferation, and renal tubular epithelial cells were neatly arranged with no vacuolar degeneration. In contrast, the T2DM group exhibited enlarged glomeruli, mesangial matrix proliferation, and renal tubular epithelial cells showing edema and vacuolar degeneration. In the HSYH and HSYL groups, the glomeruli were mildly enlarged, with less mesangial proliferation compared to the T2DM group, and reduced vacuolar degeneration in the renal tubules (Figure 4A–D).

Masson staining demonstrated that in the NC group, the glomeruli and tubular basement membranes were intact, with minimal collagen fibers (blue) visible in the interstitial area. In the T2DM group, there was significant blue collagen deposition in the mesangial region of the glomeruli, basement membrane thickening, and marked renal interstitial fibrosis, with collagen fibers surrounding the tubules. In the HSYH and HSYL groups, collagen deposition in the mesangial area of the glomeruli was reduced, and the extent of interstitial fibrosis was lessened compared to the T2DM group (Figure 4 E–H).

RAGE expression was localized to the glomeruli, renal tubular epithelial cells, and interstitial regions. In the NC group, RAGE expression was minimal, with occasional slight expression in the glomeruli or tubules. In the T2DM group, RAGE was diffusely highly expressed in the mesangial area of the glomeruli. In the HSYH and HSYL groups, RAGE staining was weaker in the glomeruli compared to the T2DM group, and the positive staining in the renal tubular epithelial cells was reduced (Figure 4I–M).

### 3.5. HSY Improved the Gut Microbiota Imbalance in T2DM Mice

16S rRNA sequencing was conducted to analyze the gut microbiota in the colonic contents of mice from the NC, T2DM, and HSY groups. α‐diversity, which describes species richness, abundance, and evenness of distribution within a community, showed no significant differences among the samples from the three groups (Figure 5A). However, β‐diversity analysis revealed distinct clustering of the three groups, with significant separation between them (Figure 5B).

Figure 516s rRNA sequencing results of the gut microbiota in mice (*n* = 6). (A) α‐diversity (Shannon index); (B) β‐diversity (PCA scatter plot); (C) Phylum‐level clustering heatmap; (D) Genus‐level clustering heatmap.

**Figure:**
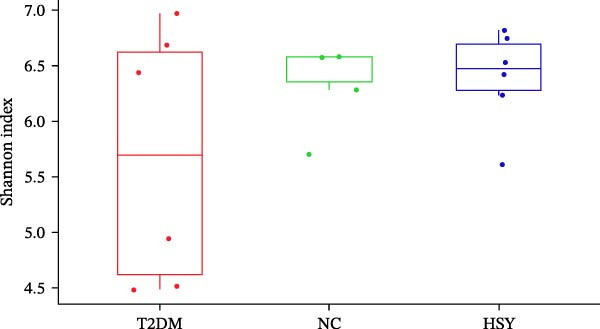
(A)

**Figure:**
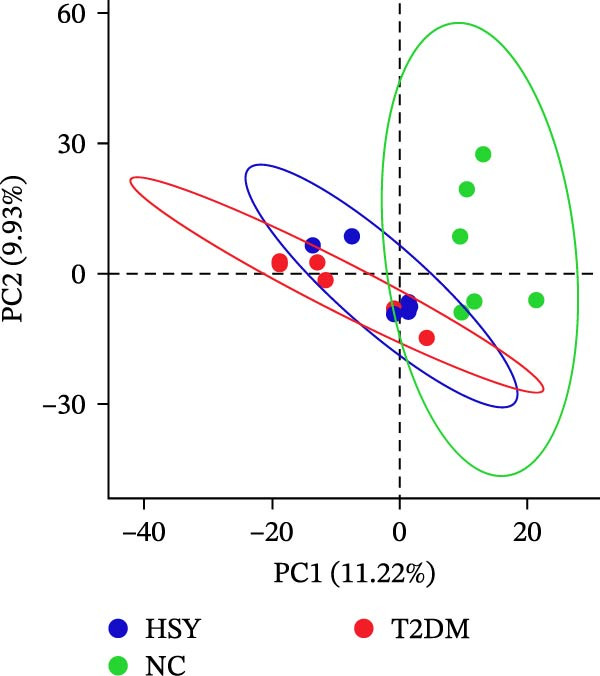
(B)

**Figure:**
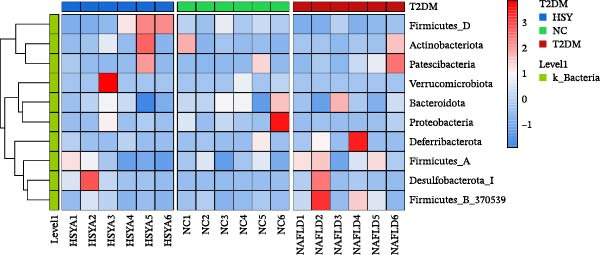
(C)

**Figure:**
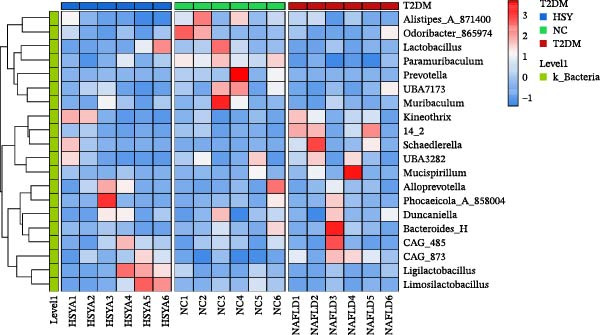
(D)

At the phylum level, HSY treatment did not induce significant changes in microbial abundance in the colon of T2DM mice (Figure 5C). At the genus level, HSY treatment resulted in an increased abundance of *Lactobacillus*, *Odoribacter*, *Ligiatobacillus*, and *Limosilactobacillus* in the intestines of T2DM mice, while the abundance of Schaedlerella was significantly decreased (*p* < 0.05) (Figure 5D).

### 3.6. HSY Corrected the Serum Metabolomics Disorder in T2DM Mice

The mechanism of HSY treatment for DKD was further investigated through non‐targeted metabolomics of mouse serum. Principal component analysis (PCA) revealed that samples from the three groups (NC, T2DM, and HSY) clustered separately, with significant separation observed in the ESI‐mode. In this mode, the HSY group was closer to the NC group than to the T2DM group, indicating that HSY treatment had a notable regulatory effect on the metabolic disorder in T2DM mice (Figure 6A). OPLS‐DA also demonstrated distinct clustering between the T2DM and HSY groups, reflecting significant changes in serum metabolomics after HSY intervention (Figure 6B). Differential metabolites regulated by HSY were identified using the criteria of VIP >1 and *p*  < 0.05 and presented in a clustering heatmap (Figure 6C,D). Further enrichment analysis using the KEGG pathway database highlighted that HSY treatment in T2DM mice influenced metabolic pathways associated with glucose and lipid metabolism, including riboflavin metabolism, linoleic acid metabolism, and starch and sucrose metabolism (Figure 7).

Figure 6The results of the non‐targeted metabolomics analysis of mouse serum (*n* = 6). The PCA plot clearly shows a significant separation between the NC group, NAFLD group, and HSY group, with HSY significantly correcting the metabolic disorder in the serum of mice from the NAFLD group (A). Based on the OPLS‐DA analysis results (B), and using the VIP > 1 and *p* < 0.05 criteria, the differential metabolites under ESI^+^ and ESI^−^ modes were identified (C and D).

**Figure:**
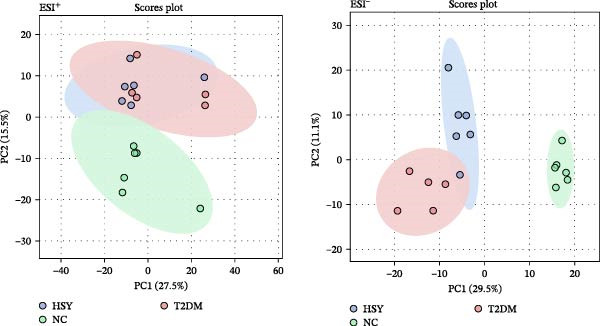
(A)

**Figure:**
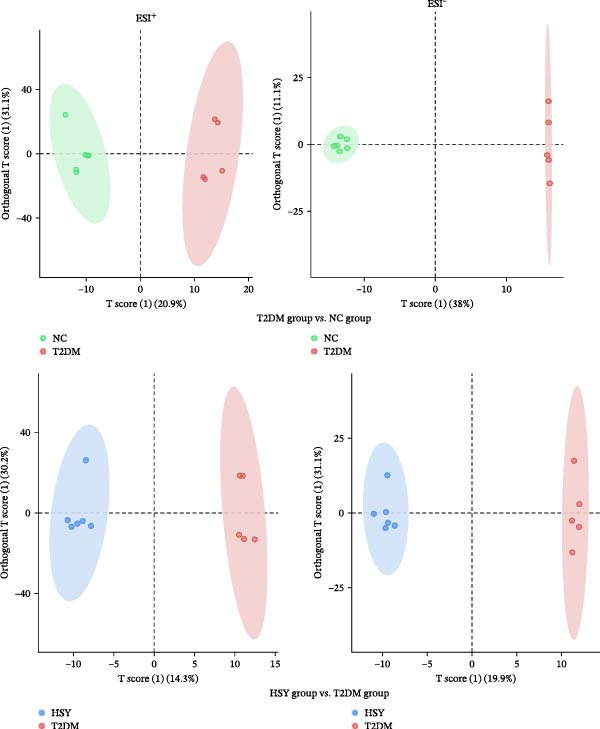
(B)

**Figure:**
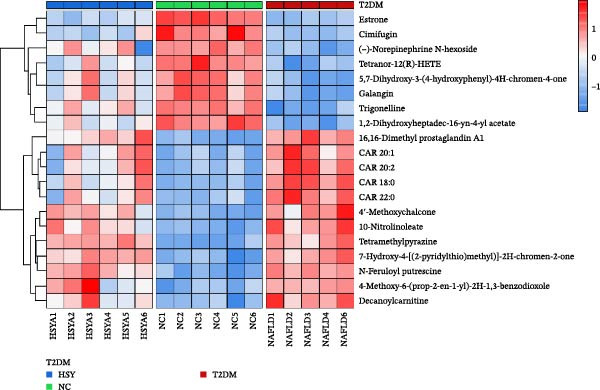
(C)

**Figure:**
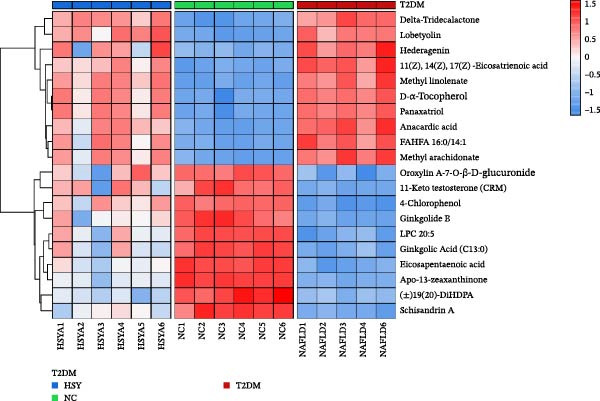
(D)

Figure 7Enrichment results of serum metabolic pathways in mice (*n* = 6). (A) In ESI^+^ mode, HSY significantly affects the serum metabolic pathways in NAFLD mice, including ① steroid hormone biosynthesis, ② caffeine metabolism, ③ starch and sucrose metabolism, ④ arginine and proline metabolism, and ④ purine metabolism. (B) In ESI^−^ mode, HSY significantly affects the serum metabolic pathways in NAFLD mice, including ① butyrate metabolism, ② niacin and nicotinamide metabolism, ③ unsaturated fatty acid biosynthesis, and ④ cytochrome P450 metabolism of exogenous substances.

**Figure:**
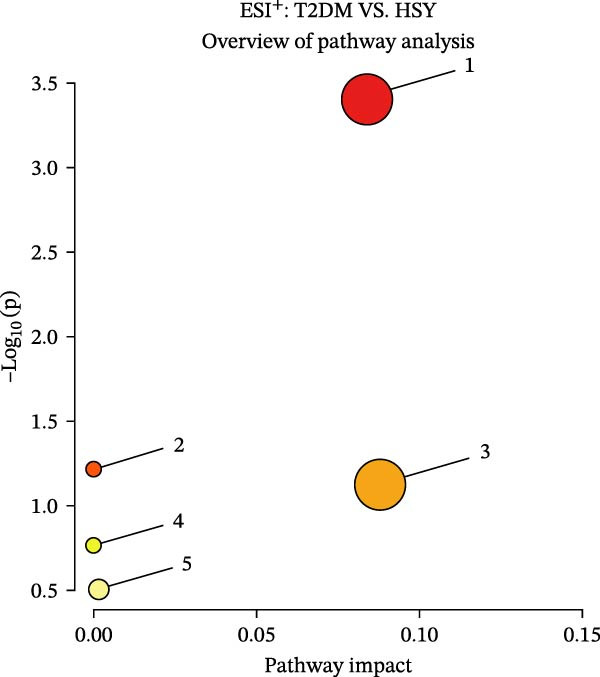
(A)

**Figure:**
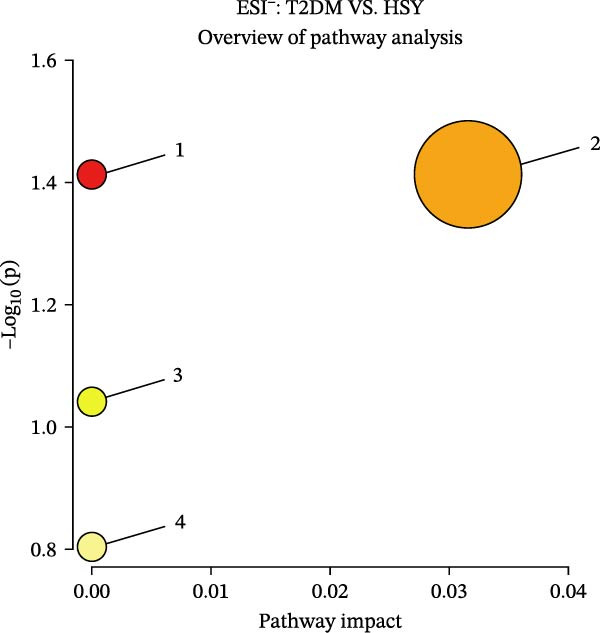
(B)

### 3.7. Spearman Correlation Analysis of Gut Microbiota and Serum Metabolomics

**Figure 8:**
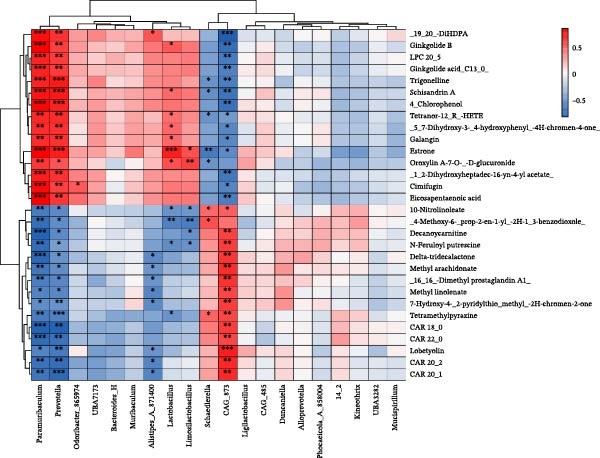
Spearman correlation analysis of gut microbiota and serum metabolomics.

The abundance of *Prevotella* and *Muribaculum* in the mouse gut correlates positively with the serum concentrations of DiHDPA, estrone, and eicosapentaenoic acid. Similarly, the abundance of *Lactobacillus* is positively correlated with the serum concentration of estrone. In contrast, a negative correlation exists between the serum concentration of estrone and the abundance of *Schaedlerella* in the gut (Figure 8).

## 4. Discussion

The present study provides substantial mechanistic insights into the therapeutic effects of HSY on DKD through an innovative multi‐omics approach that integrates network pharmacology, gut microbiome analysis, and serum metabolomics. While previous investigations have established HSY’s anti‐inflammatory and antioxidant properties in isolated contexts, our work represents the first comprehensive exploration of its renoprotective effects mediated through gut‐kidney axis modulation and metabolic pathway regulation. The study delineates several novel aspects that distinguish it from prior research and significantly advance our understanding of HSY’s therapeutic potential.

A hallmark of DKD progression is the excessive accumulation of AGEs, which exacerbate renal damage through interactions with the receptor for AGEs (RAGE) [24]. The AGE–RAGE axis activates downstream pro‐inflammatory pathways, including the NLRP3/Caspase‐1 pathway, which further amplifies oxidative stress and cytokine production (e.g., TNF‐α, IL‐1β) [25–27]. In this study, HSY administration significantly suppressed renal RAGE expression, accompanied by reductions in TNF‐α and IL‐1β levels, suggesting its ability to disrupt AGE–RAGE‐mediated inflammatory signaling. This aligns with prior research indicating that RAGE blockade mitigates macrophage infiltration and tubulointerstitial fibrosis in diabetic kidneys [28, 29]. Additionally, HSY’s quinone‐chalcone scaffold may directly interfere with AGE–RAGE binding, as similar polyphenolic compounds have been shown to competitively inhibit RAGE–ligand interactions [30–32].

Notably, HSY restored renal antioxidant defenses, as evidenced by elevated activities of SOD, GSH, and CAT. AGE–RAGE activation is known to deplete endogenous antioxidants through NADPH oxidase‐driven ROS overproduction [33, 34]. By mitigating RAGE overexpression, HSY likely interrupts this vicious cycle, preserving redox balance. The compound’s inherent radical‐scavenging properties may complement RAGE inhibition, providing dual antioxidant effects—neutralizing existing ROS and preventing their generation [35, 36]. This mechanism may account for the superior renoprotective effects observed with HSY, surpassing the efficacy of single‐target antioxidants.

Network pharmacology analysis further supports AGE–RAGE as a key target, although HSY’s broad spectrum of effects likely involves additional pathways. For instance, improved lipid profiles (reduced triglycerides and cholesterol) may reflect AMPK activation, a known target of HSY in metabolic disorders, which indirectly suppresses AGE formation by enhancing glucose metabolism [37, 38]. Furthermore, the reduction in serum urea nitrogen and creatinine underscores HSY’s role in preserving glomerular filtration, possibly through anti‐apoptotic effects on podocytes, a cell type particularly vulnerable to AGE‐induced damage [39, 40].

While the study emphasizes the AGE–RAGE pathway, limitations include the need for RAGE‐knockout models to confirm pathway specificity and omics‐based profiling to uncover off‐target effects. Additionally, further exploration of HSY’s pharmacokinetics, especially its renal bioavailability, is necessary to optimize dosing strategies. In conclusion, HSY mitigates DKD by targeting the AGE–RAGE axis, effectively quenching inflammatory and oxidative cascades. This work positions HSY as a promising multi‐target agent for diabetic complications, warranting clinical investigation alongside conventional therapies.

16S rRNA sequencing revealed that HSY induced the enrichment of SCFA‐producing genera (Lactobacillus and Alloprevotella), which was accompanied by increased fecal levels of acetate, propionate, and butyrate [41, 42]. SCFAs inhibit inflammation through three primary mechanisms. Butyrate acts as a histone deacetylase (HDAC) inhibitor, enhancing histone acetylation to promote Foxp3 expression and regulatory T‐cell differentiation, thereby suppressing NF‐κB‐driven TNF‐α production [43–46]. Acetate and propionate bind to GPR43/41, inhibiting NLRP3 inflammasome activation in renal macrophages and reducing IL‐1β maturation [47–49]. Furthermore, butyrate upregulates tight junction proteins, reducing systemic endotoxemia (e.g., lipopolysaccharide), a key mediator of renal RAGE overexpression [50, 51].

HSY’s microbial remodeling likely works in synergy with its direct anti‐RAGE activity to disrupt the gut‐kidney inflammatory axis. For instance, Lactobacillus species not only produce SCFAs but also secrete indole derivatives that activate aryl hydrocarbon receptors (AhR), further suppressing renal IL‐6 and MCP‐1 expression [52–54]. This dual anti‐inflammatory action (SCFAs plus direct RAGE inhibition) sets HSY apart from probiotics, which lack intrinsic RAGE‐targeting capabilities.

Comprehensive serum metabolomic profiling revealed that HSY modulates pathways related to inflammation and redox homeostasis. Non‐targeted metabolomics analysis identified significant perturbations in inflammation‐associated and oxidative stress‐related metabolic pathways following HSY intervention. Of particular interest was the identification of 11‐Keto Testosterone (11‐KT), an androgen derivative with potent anti‐fibrotic effects through targeted inhibition of the TGF‐β/Smad3 signaling axis in renal fibroblasts [9]. Mechanistic studies indicate that 11‐KT exerts its therapeutic effect by competitively binding to Smad3 phosphorylation sites, disrupting the canonical fibrotic signaling cascade.

Additionally, Norepinephrine N‐hexoside, a novel redox‐modulating metabolite with dual regulatory functions, was identified. This compound appears to regulate cellular antioxidant responses through β‐adrenergic receptor‐mediated upregulation of phase II detoxification enzymes, particularly heme oxygenase‐1 (HO‐1) [10]. The observed induction of HO‐1 correlates with enhanced nuclear translocation of Nrf2 and subsequent activation of the antioxidant response element (ARE), suggesting a sophisticated epigenetic regulatory mechanism underlying its oxidative stress attenuation properties.

These metabolomic findings collectively highlight HSY’s multifaceted therapeutic potential, simultaneously modulating inflammatory signaling through steroid hormone pathway modulation while activating endogenous antioxidant defenses. The synergistic interactions among these identified metabolites provide new insights into the molecular mechanisms underlying HSY’s renal protective effects, establishing a metabolic framework for future therapeutic strategies in fibrotic disorders.

Metabolomic profiling identified two critical lipid mediators—Eicosatrienoic acid and Methyl arachidonate—as key upstream regulators of pro‐inflammatory eicosanoid biosynthesis. These ω−6 polyunsaturated fatty acids act as rate‐limiting substrates for cyclooxygenase (COX) and lipoxygenase (LOX)‐mediated production of leukotriene B4 (LTB4) and prostaglandin E2 (PGE2), potent chemoattractants that drive renal leukocyte infiltration. The HSY‐induced depletion of these precursors mechanistically explains the significant improvement in renal injury and fibrosis observed in T2DM mice, suggesting targeted suppression of the arachidonic acid cascade—a hallmark of DKD progression.

Simultaneously, decanoylcarnitine emerged as a diagnostic biomarker of mitochondrial β‐oxidation insufficiency, with its accumulation reflecting electron transport chain uncoupling and subsequent lipotoxic ROS overproduction. HSY treatment led to a significant decrease in acyl‐carnitine profiles, indicating restored fatty acid flux through CPT1A‐mediated mitochondrial shuttling. This metabolic rewiring coincided with reduced malondialdehyde levels, directly linking mitochondrial rehabilitation to attenuated lipid peroxidation cascades.

While these findings highlight HSY’s multi‐organ protective effects, key questions remain: The causal relationship between specific microbial taxa (e.g., *Limosilactobacillus*) and SCFA production requires validation via fecal microbiota transplantation (FMT). Additionally, clinical trials in DN individuals with defined gut dysbiosis phenotypes are essential to evaluate the reproducibility of HSY’s therapeutic effects across diverse populations.

## 5. Conclusion

The study provides compelling evidence for HSY’s dual action targeting both AGE‐RAGE signaling inhibition and oxidative stress mitigation. While RAGE suppression has been reported for other natural compounds, HSY demonstrates superior efficacy through its ability to simultaneously preserve renal antioxidant defenses (GSH, SOD, CAT) and interfere with AGE‐RAGE‐mediated inflammatory cascades (NF‐κB, NLRP3 inflammasome). Importantly, these direct molecular effects synergize with HSY’s microbiota‐mediated actions, representing a multi‐modal therapeutic approach not achievable with single‐target agents.

**Figure 9:**
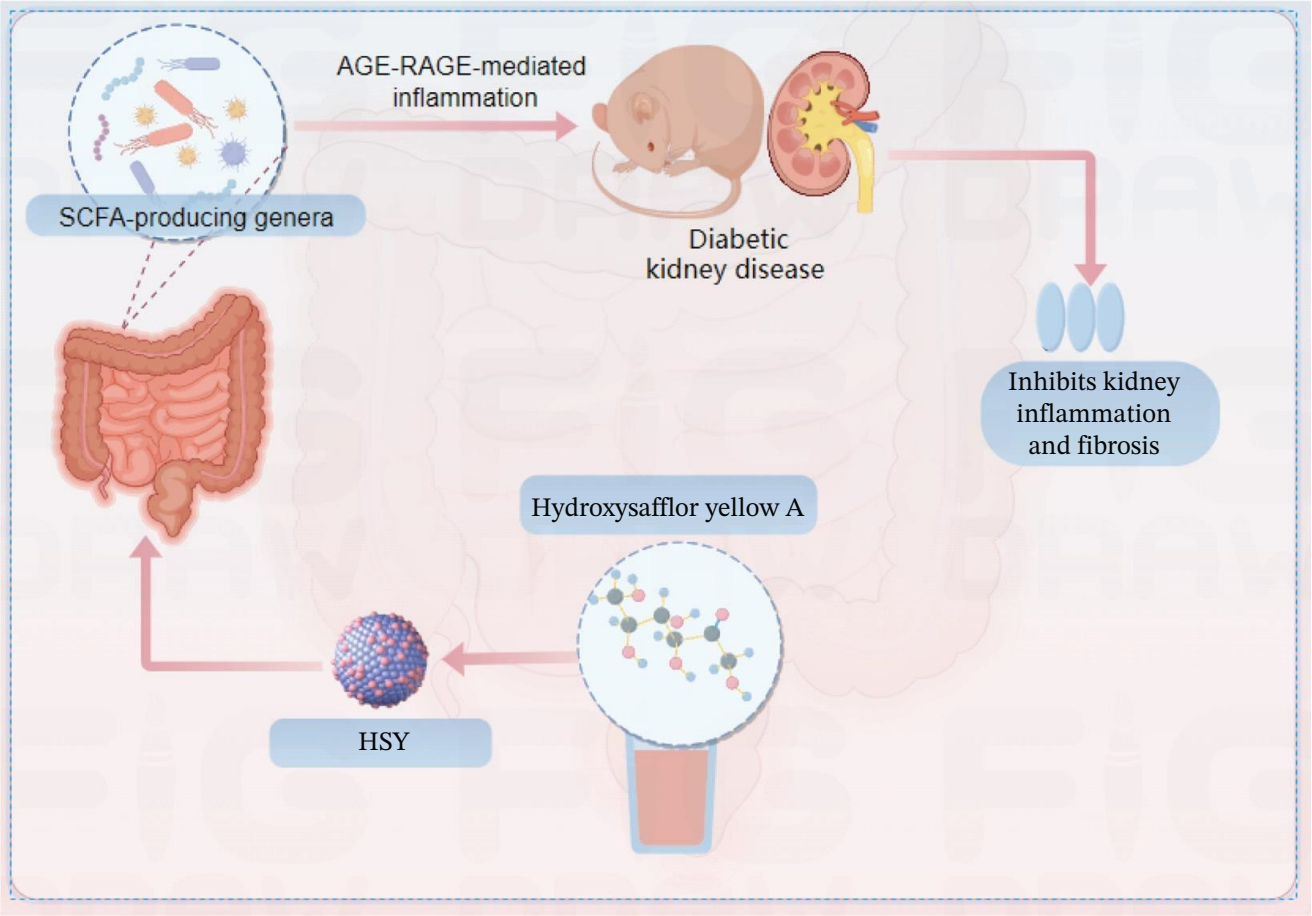
Mechanism diagram of HSY improving diabetic kidney disease by modulating gut microbiota to inhibit the AGE−RAGE pathway and subsequent inflammatory responses.

From a translational perspective, our findings suggest HSY’s potential as a comprehensive DKD therapeutic capable of addressing multiple pathological aspects including inflammation, oxidative stress, metabolic dysregulation, and gut dysbiosis simultaneously. The identification of specific microbial signatures (e.g., *Lactobacillus* enrichment) and metabolic biomarkers (e.g., altered steroid hormone pathways) associated with HSY treatment provides measurable targets for future clinical studies and personalized therapeutic approaches (Figure 9).

## Author Contributions

Conceptualization: Pingping Wang and Xinyu Liu. Methodology: Xinyu Liu, Wen Sun, and Xueyun Dong. Software: Jiajun Tan. Validation: Min Chen and Jiayuan He. Formal analysis: Asmaa Ali. Investigation: Jiayuan He, Liang Wu, and Keke Shao. Resources: Pingping Wang. Data curation: Liang Wu. Writing – original draft preparation: Pingping Wang and Xinyu Liu. Writing – review and editing: Asmaa Ali, Liang Wu, and Keke Shao. Visualization: Wen Sun, Xueyun Dong, and Jiajun Tan. Supervision: Asmaa Ali, Liang Wu, and Keke Shao. Funding acquisition: Liang Wu.

## Funding

This work was supported by Zhenjiang Science and Technology Innovation Fund (Key R&D Program—Social Development) Project (SH2023073), Scientific Research Project of Yancheng Municipal Health Commission (YK2024116), and Project of Jiangsu Provincial Science and Technology Development Plan for Traditional Chinese Medicine (MS2022126).

## Ethics Statement

The study was conducted in accordance with the National Institutes of Health guide for the care and use of Laboratory animals (NIH Publications No. 8023, revised 1978), and approved by the Ethics Committee of the Jiangsu University (protocol code UJS‐IACUC‐AP‐2023011012 and date of approval: January 2023).

## Conflicts of Interest

The authors declare no conflicts of interest.

## Supporting Information

Additional supporting information can be found online in the Supporting Information section.

## Supporting information


**Supporting Information** S1: Network Pharmacology Analysis. S2: Construction of T2DM mouse model and experimental grouping. S3: Detection of serum biochemical indicators and renal tissue oxidative stress indicators. S4: The qRT‐PCR assay; S5: 16s rRNA sequencing of gut microbiota and serum untargeted metabolomics analysis. Table S1: qPCR primers sequence.

## Data Availability

The data presented in this study are available upon request from the corresponding authors.
