# Supplementary material for: Network Pharmacology Combined With Gut Microbiome and Serum Metabolomics Reveals the Therapeutic Mechanisms of Hydroxysafflor Yellow A in Diabetic Kidney Disease
Source: J Diabetes Res. 2026 Apr 1;2026:2131566. doi: 10.1155/jdr/2131566 (PMC13045261; doi:10.1155/jdr/2131566)
Supplement: Supplementary file 1 — Supporting Information S1: Network Pharmacology Analysis. S2: Construction of T2DM mouse model and experimental grouping. S3: Detection of serum biochemical indicators and renal tissue oxidative stress indicators. S4: The qRT‐PCR assay; S5: 16s rRNA sequencing of gut microbiota and serum untargeted metabolomics analysis. Table S1: qPCR primers sequence. [file JDR-2026-2131566-s001.docx]

**Supplement**

*S1: Network Pharmacology Analysis.* The HSY structure obtained from the PubChem database (https://pubchem.ncbi.nlm.nih.gov/) was imported into the Searesults database (https://sea.bkslab.org/), SwissTargetPrediction database (http://www.swisstargetprediction.ch/), Targets SUP-PRED database (https://prediction.charite.de/), and the cbligand database (https://www.cbligand.org/) for drug target prediction.

Disease-related targets were identified by searching "type 2 diabetes mellitus" in the GeneCards database (https://www.genecards.org/), OMIM database (https://omim.org/), and TTD database (https://db.idrblab.net/). A Venn diagram was created to visualize the overlap between component targets and disease targets. The intersection of drug action targets and disease targets was imported into the String database (https://cn.string-db.org/). The search results were exported in "tsv" format and imported into Cytoscape 3.9.0 software. Using the CytoNCA plugin, network topology parameters were filtered to identify core targets for HSY treatment of T2DM based on degree value. The intersected targets were uploaded into the DAVID database (https://davidbioinformatics.nih.gov/) for enrichment analysis. The results were visualized to include biological processes, molecular functions, and cellular components from the GO of the target genes, along with the key signaling pathways of HSY’s action targets identified through KEGG data (*P* < 0.05).

*S2. Construction of T2DM Mouse Model and Experimental Grouping.* Twenty-two to twenty-six healthy male ICR mice (Wukong Biotechnology Co., LTD, Nanjing, China) were used to establish the T2DM model. The mice were randomly assigned to four groups: a normal control group (NC), a diabetes group (T2DM), a high-dose HSYA group (HSYH, 10 mg/kg), and a low-dose HSYA group (HSYL, 5 mg/kg), with six mice per group. As described by Yan et al., six-week-old healthy ICR mice were fed a high-fat diet (60% of energy from fat) for two weeks. Starting in week 3, streptozotocin was administered *via* intraperitoneal injection for three consecutive days (50 mg/kg/day, Merck, USA). At the beginning of the fourth week, fasting blood glucose levels were measured, and mice with fasting blood glucose levels exceeding 11.1 mmol/L were considered successfully diabetic. These mice continued the high-fat diet until the end of week 12. From the first day of the experiment, the HSYH and HSYL groups received HSY by gavage (Shanghai Shifeng Biological Co. Ltd, Shanghai, China). At the end of week 12, the experiment concluded, and samples were collected for further analysis. After completing the experimental period, the mice were euthanized by intraperitoneal injection of Urethan (Sigma-Aldrich, St. Louis, MO, USA, 700 mg/kg). Serum, colon contents, and kidney tissues were collected for subsequent research.

*S3. Detection of Serum Biochemical Indicators and Renal Tissue Oxidative Stress Indicators.* The concentrations of glucose (F006-1-1), blood urea nitrogen (BUN, C013-2-1), creatinine (CRE, C011-2-1), total cholesterol (TC, A111-1), triglycerides (TG, A110-1), high-density lipoprotein cholesterol (HDL-C, A112-1), and low-density lipoprotein cholesterol (LDL-C, A113-1) in mouse serum were measured using kits from Jiancheng Bioengineering Institute (Nanjing, China). Additionally, the activity/concentration of reduced glutathione (GSH, A006-1-1), total superoxide dismutase (T-SOD, A001-1), and catalase (CAT, A007-1-1) in mouse kidney tissue were assessed. The advanced glycation end products (AGEs, E-EL-0052c) detection kit was supplied by Meimian Bioengineering Institute (Yancheng, China). All procedures were strictly followed according to the kit instructions.

*S4. The qRT-PCR Assay.* The qRT-PCR was performed to determine the mRNA expression levels of TNF-α, IL-1β, and RAGE in mouse kidney tissue. All reagents were provided by Vazyme (Nanjing, China). Total RNA was extracted from mouse kidney tissue using the RNA-easy Isolation Reagent (R701-01). cDNA was synthesized using the HiScript III RT SuperMix for qPCR (+gDNA wiper) kit (R323-01), and subsequent qPCR was performed with the AceQ Universal SYBR qPCR Master Mix kit (Q711-02). Primer sequences are listed in Table S1, and relative gene expression levels were calculated using the 2^−ΔΔCT^ method.

**TABLE S1 qPCR primers sequence**

| Genes | Upstream primer | Downstream primer |
| --- | --- | --- |
| TNF-α | CATCTTCTCAAAATTCGAGTGACAA | TGGGAGTAGACAAGGTACAACCC |
| IL-1β | GAAATGCCACCTTTTGACAGTG | TGGATGCTCTCATCAGGACAG |
| RAGE | AGTCCAAGAGCGGAACCTAC | CTCGGCCATAGTTTCCACTC |
| β-actin | GGCTGTATTCCCCTCCATCG | CCAGTTGGTAACAATGCCATGT |

*S5. 16s rRNA Sequencing of Gut Microbiota and Serum Untargeted Metabolomics Analysis.* The analysis of the gut microbiota and untargeted serum metabolomics in mice was performed by Ekemo Tech Group Co., Ltd. (Shenzhen, China). Colonic contents were collected for bacterial 16S rRNA gene sequencing. The obtained gene sequences were annotated for species identification and subjected to bioinformatics analysis, including α-diversity (Shannon index) to assess the quantity and relative abundance of gut microbiota, β-diversity (PCoA) to evaluate model establishment and predictability, and a clustering heatmap to explore the impact of HSYA on the gut microbiota structure.

Non-targeted metabolomics data from mouse serum were analyzed using PCA and OPLS-DA to assess the effectiveness of model development. One-way analysis of variance was performed to compare differences in metabolite levels between groups. Differential metabolites were identified using VIP > 1.0 and *P* < 0.05 as selection criteria. These metabolites were further analyzed using the KEGG database for metabolic pathway analysis *via* the MetaboAnalyst 6.0 platform (https://www.metaboanalyst.ca/).
